# Physiological and Biochemical Response of *Alternanthera bettzickiana* (Regel) G. Nicholson under Acetic Acid Assisted Phytoextraction of Lead

**DOI:** 10.3390/plants9091084

**Published:** 2020-08-24

**Authors:** Urousa Latif, Mujahid Farid, Muhammad Rizwan, Hafiz Khuzama Ishaq, Sheharyaar Farid, Shafaqat Ali, Mohamed A. El-Sheikh, Mohammed Nasser Alyemeni, Leonard Wijaya

**Affiliations:** 1 Department of Environmental Sciences, University of Gujrat, Hafiz Hayat Campus, Gujrat 50700, Pakistan; uroosalatif9@gmail.com (U.L.); mujahid.farid@uog.edu.pk (M.F.); 18071761-012@uog.edu.pk (H.K.I.); 2 Department of Environmental Sciences and Engineering, Government College University, Faisalabad 38000, Pakistan; mrizwan@gcuf.edu.pk (M.R.); sherryfarid01@gmail.com (S.F.); 3 Department of Biological Sciences and Technology, China Medical University, Taichung 40402, Taiwan; 4 Department of Botany and Microbiology, College of Science, King Saud University, Riyadh 11451, Saudi Arabia; melsheikh@ksu.edu.sa (M.A.E.-S.); mnyemeni@ksu.edu.sa (M.N.A.); leon077@gmail.com (L.W.)

**Keywords:** accumulation, bioavailability, enzymatic activities, phytoextraction, reactive oxygen species

## Abstract

Heavy metals (HMs) stress causes severe damage to physiology and biochemistry of plant species leading to stunted growth and low yield. Phytoremediation via phytoextraction, a viable low-cost and environment-friendly alternative to other techniques that are often too expensive, impractical and hazardous. However, phytoextraction potential, physiological and biochemical response of various plant species against HMs stress is not fully understood. Among other HMs, lead (Pb) is an inorganic pollutant with deleterious biotic effects. Bioavailability and mobility of the Pb can be enhanced by addition of organic acids. A pot scale experiment was done to assess the effects of Pb on *Alternanthera bettzickiana* (Regel) G. Nicholson and its ability to accumulate Pb with or without acetic acid (AA). The Results showed that Pb caused significant damage in *A. bettzickiana*, and its ecotoxicity was evident from increased levels of lipid peroxidation up to 107% under Pb stress. The significant decrease in plant height (32%), root length (21%), leaf area (38%) and number of leaves per plant (46%) was observed. On the other hand, application of AA to Pb stressed plants reduced the oxidative damage by further enhancing the activities of ascorbate peroxidase (APX) and catalases (CAT) up to 16% and 21% respectively. Moreover, addition of AA significantly improved plant total chlorophylls (15%) and carotenoids (50%). The application of AA also promoted Pb accumulation in leaf, stem and roots up to 70%, 65% and 66% respectively. This research concluded that AA has the ability to enhance the phytoextraction of Pb and support the plant growth and physiology under Pb stress condition.

## 1. Introduction

Human life, surrounded by luxuries and technological advances, has produced a variety of wastes including organic and inorganic pollutants that have deleterious effects on the biotic (fauna and flora) and abiotic (air, water, and soil) components of the environment [1]. Among these pollutants, HMs are highly persistent and bioaccumulate in the environment due to their non-biodegradable nature [2]. Various studies have been performed to understand the toxicity of Pb in the ecosystem [3]. In soil, Pb is highly persistent and potentially poisonous, even at very low concentrations [4]. These anthropogenic activities severely harm the biosphere, and particularly human health and other ecological systems. Pb has the ability to change various metabolic pathways in many plants as well as decrease nutrient uptake and the rate of transpiration [2,4]. The permissible limit approved by the Environment Protection Agency, USA (US-EPA) for Pb is 50 mg L^−1^ in aquatic environments; this limit approved by Environment Protection Agency of Pakistan (Pak-EPA) is 0.5 mg L^−1^ for land water, soil, and sea for the protection of human health [5].

In plants, HMs concentration is the major reason for oxidative stress generated by reactive oxygen species (ROS) [6]. These ROS can overcome cells’ essential antioxidant systems and lead to cell death [7]. Chlorophylls are more susceptible to the negative impact of HMs compared to carotenoids [8]. Heavy metals decrease cell viability by severely affecting nascent protein homeostasis through interfering with the folding process and the aggregation of nascent [9].The plant biochemical response, including antioxidant enzymes such as superoxide dismutase (SOD), ascorbate peroxidase (APX), and catalase (CAT), increases its activities under HMs stress to overcome enhanced lipid peroxidation [10]. Heavy metals affect living organisms by accumulating in their fatty tissues (bioaccumulation) and pass from one trophic level to the next trophic level (biomagnification) [6,7].

Many physical approaches are used for treating polluted soil including capping, deep burial, and soil excavation, along with chemical methods techniques that also affect the physical, chemical, and biological properties of soil, and making it useless for the growth of plants [11]. Keeping in view these constraints, the branch of bioremediation technique known as phytoremediation, further categorized as phytoextraction, is considered an attractive substitute for soil remediation, because it is one of the cheapest and most ecofriendly method compared with other physical and chemical techniques [7,12]. Halophytes are naturally capable of living in dry, rough, and saline environmental conditions and have the ability to absorb harmful HMs [13]. *A. bettzickiana* is perennial hardy plant that is capable of growing in highly saline, dry, and hot environments and only a few studies have evaluated the phytoremediation potential of *A. bettzickiana* for Pb, Cd, and Cr [14,15]. The physiological and biochemical responses of *A. bettzickiana* under HMs stress have not been evaluated extensively in the literature.

Different organic acids, for example, oxalic acid, citric acid, and acetic acid, are used as chelating agents in the phytoextraction process to enhance the bioavailability of HMs for plant uptake, and these acids play important roles in the rhizosphere [16]. These organic acids are typically involved in many processes that control the physiochemical properties of the soil and help plants to uptake insoluble, deficient, and unavailable nutrients from the soil [17]. These acids perform a number of functions such as maintenance of the carbon cycle, breaking down complex nutrients, providing nutrients to microbes, and detoxifying organic and inorganic pollutants [18]. These organic acids have the ability to dissolve the complex nutrients in the soil and promote three major processes: exchange reaction, chelation, and acidification, resulting in enhanced solubility of HMs in the soils [19,20].

The objectives of this study were (a) to observe the influence of Pb concentration on growth characteristics of *A. bettzickiana*, (b) to measure the effect of AA on the physiology and growth of *A. bettzickiana* against Pb stress, and (c) to evaluate the phytoextraction potential of *A. bettzickiana* for Pb with AA amendment.

## 2. Results

### 2.1. Agronomic Traits

Significant variation was found in the agronomic traits of *A. bettzickiana* treated with different concentration of Pb, including plant height, root length, leaf area, number of leaves per plant, and fresh and dry biomass (leaf, stem, and root), as shown in Table 1. Increasing concentrations of Pb significantly affected the plants’ agronomic traits. The maximum decrease in agronomic traits was measured at the highest concentration (10 mM) compared to the rest of the treatments.

Pb at (10 mM) significantly reduced plant height, root length, leaf area, and the number of leaves per plant by 32%, 21%, 38%, and 46%, respectively, compared to the control treatments. Similarly, fresh and dry biomass of root, stem, and leaf declined by 42%, 67%, and 46% and 45%, 53%, and 70%, respectively, in comparison to the control. However, the addition of AA significantly alleviated Pb-induced morphological damage by modulating the morphology of the plants. This growth-promoting role of AA under Pb stress is also evident in Table 1 and Table 2. The application of AA (2.5 mM) alone or with Pb (2.5, 5, 7.5, and 10 mM) significantly enhanced the agronomic traits of plants. The maximum increase in the growth of plant height (32%), root length (29%), leaf area (47%), and number of leaves per plant (37%) was measured compared with the rest of the treatments. Similar results were observed in fresh and dry biomass of leaf, stem, and root: 9–41%, 5–63%, and 38–65%, and 14–54%, 14–65%, and 17–40% respectively under combined application of AA and Pb (0–10 mM).

### 2.2. Photosynthetic Pigments

**Figure 1:**
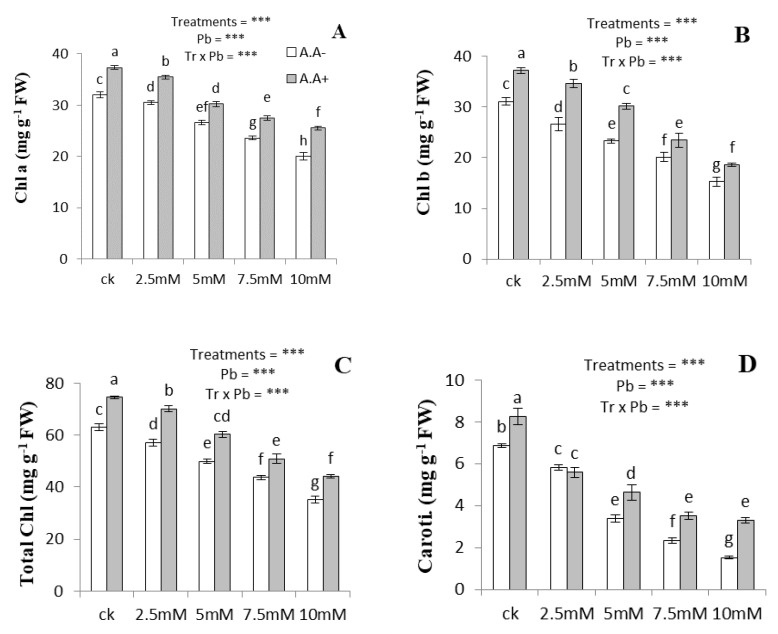
Effect of Pb and AA on chlorophyll a (**A**), chlorophyll b (**B**), total chlorophylls (**C**) and carotenoids (**D**) in *A. bettzickiana* grown in soil with increasing Pb (0, 2.5, 5, 7.5 and 10 mM) and AA concentrations (0 and 2.5 mM). Values are mean of three replicates ± S.D. Different lowercase letters indicate significant differences between treatments at *p* < 0.05; and *** indicate significance at the *p* < 0.01 level.

The increasing concentration of applied Pb caused significant reductions in carotenoids and chlorophylls contents compared to the control (Figure 1). At 10 mM Pb, the contents of Chl a, Chl b, total Chl, and carotenoids decreased by 37%, 50%, 43%, and 77%, respectively. The AA amendment reduced Pb-induced toxic effects by improving the carotenoids and chlorophyll contents of plants. A significant improvement was observed in Chl a (13–28%), Chl b (16–30%), total Chl (7–15%), and carotenoids (7–50%) contents under combined application of Pb and AA as compared to Pb treated only plant.

### 2.3. Antioxidant Enzymatic Activities and MDA Production

**Figure 2:**
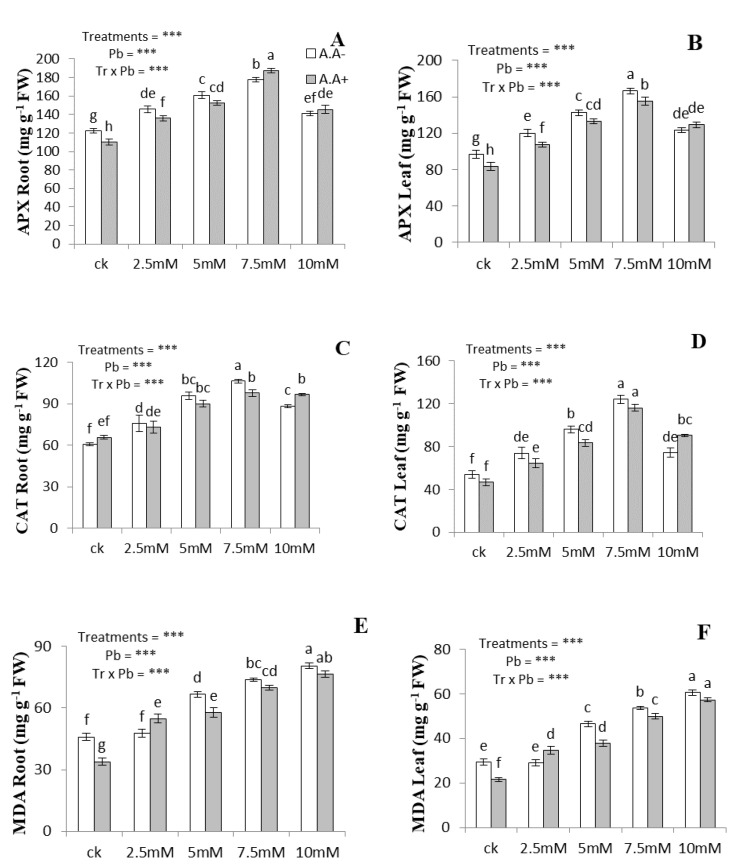
Effect of Pb and AA on APX in roots (**A**), APX in leaves (**B**), CAT in root (**C**), CAT in leaves (**D**), MDA in root (**E**) and MDA in leaves (**F**) of *A. bettzickiana* grown in soil with increasing Pb concentrations (0, 2.5, 5, 7.5 and 10 mM) and AA (0 and 2.5 mM). Values are mean of three replicates ± S.D. Different lowercase letters indicate significant differences between treatments at *p* < 0.05; and *** indicate significance at the *p* < 0.01 level.

The antioxidant enzymatic activities, including APX and CAT, in both roots and leaves were measured along with the production of MDA under Pb and AA applications (Figure 2). The maximum increases in the activities of APX and CAT were observed at Pb (7.5 mM) both in roots (28% and 46%, respectively) and leaves (47% and 59%, respectively). The activities of these enzymes tended to decrease both in roots and leaves at 10 mM Pb. A significant increase in the production of MDA was observed at 10 mM Pb both in roots and leaves at 75% and 107%, respectively. The addition of AA further elevated the activities of antioxidant enzymes under Pb and AA combined treatment. The highest values of antioxidant enzymatic activities were recorded both in leaves and roots under the combined application of AA and Pb (7.5 mM). Under combined application of Pb and AA, the APX and CAT activities increased both in roots (3–11% and 2–9%, respectively) and leaves (4–16% and 6–21%, respectively). The addition of AA under Pb stress significantly decreased MDA contents both in roots and leaves by 6–26% and 5–26%, respectively.

### 2.4. Soluble Protein and SPAD Value

**Figure 3:**
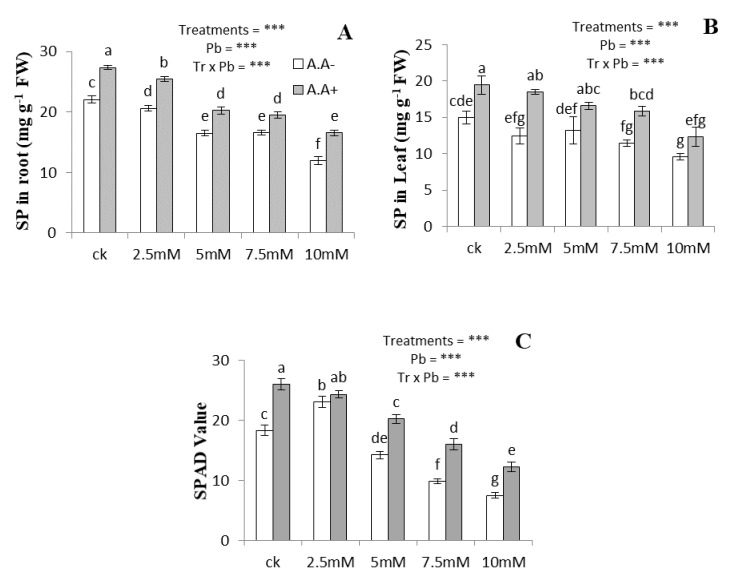
Effect of Pb and acetic acid on soluble proteins in roots (**A**), soluble proteins in leaves (**B**) and SPAD value (**C**) of *Alternanthera bettzickiana* grown in soil with increasing Pb concentrations (0, 2.5, 5, 7.5 and 10 mM) and AA (0 and 2.5 mM). Values are mean of three replicates ± S.D. Different lowercase letters indicate significant differences between treatments at *p* < 0.05; and *** indicate significance at the *p* < 0.01 level.

The soluble protein contents (root and leaf) and SPAD values of the plants declined with increasing Pb concentrations compared to the control (Figure 3). At 10 mM Pb, the protein contents in leaves and roots decreased by 36% and 45%, respectively, compared to the control. Similarly, SPAD values decreased by 60% at 10 mM Pb, respectively. The application of AA resulted in significant improvements in SPAD value and protein contents both in roots and leaves under Pb stress. The AA increased soluble protein contents both in roots and leaves by 21–37% and 25–48%, respectively. Likewise, the addition of AA increased the SPAD value by 12–63% under Pb stress.

### 2.5. Lead Concentration, Accumulation and Translocation Factor

Increasing the concentration of Pb (2.5, 5, 7.5, and 10 mM) significantly increased Pb uptake and accumulation in the leaves, stems, and roots of plants (Table 3). Trace levels of Pb were found in untreated plants, which might have been due to the presence of background Pb in the soil. The highest accumulations and concentrations of Pb were recorded at 10 mM Pb along with the application of AA in leaves, stems, and roots by 239%, 90%, 266% and 548%, 427% and 517%, respectively, compared to 2.5 mM Pb-treated plants. The addition of AA to Pb-stressed plants significantly enhanced accumulation and concentration of Pb in leaves, stems, and roots by 61–240%, 68–115%, and 44–190%, and 20–196%, 14–120%, and 12–118%, respectively. In the present study, the root–shoot translocation factor (TF) was less than but near to 1. The maximum translocation factor was observed at 2.5 mM Pb + 5 mM AA treatment at 0.91, followed by 0.87 at 5 mM Pb only.

## 3. Discussion

### 3.1. Agronomic Traits under Pb and AA Application

In plants, HMs ecotoxicity depends on various factors such as HMs concentration, exposure period, plant species, and genotype [21]. Pb interferes with plant metabolic processes, leading to deterioration of growth and development of plants [22], and particularly to reduction in photosynthesis and protein synthesis and destruction at the cellular and subcellular levels [23]. Similar toxic effects of Pb were reported in *Brassica napus* [24] and *A. bettzickiana* [15]. Similar to other organic acids, the AA growth-promoting effect is confirmed in the literature where organic acids such as citric acid (2.5, 5, and 10 mM), glutamic acid (2.5 and 5 mM), and ascorbic acid (5 mM) were used for *Brassica napus,* sunflower [24,25], *Lemna minor* L. [26], and *Solanum nigrum* L. [27], respectively, in the presence of HMs stress.

### 3.2. Chlorophyll and Carotenoids

Pb stress promoted negative effects on the transpiration rate and net photosynthetic efficiency of plants with reduced chlorophyll contents. However, the addition of AA significantly improved these contents. The literature revealed that Pb stress disturbs chloroplast, photosynthetic pigments, and protein complexes due to the increase in the activity of chlorophyllase under HMs stress [28,29]. The role of AA in promoting the photosynthetic rate occurs due to increased chlorophyll contents [30]. A similar promoting role of indole-3-acetic acid was reported in *S. nigrum* by Ji et al. [31].

### 3.3. Oxidative Stress and Antioxidant Enzymes

Ecotoxicity of Pb in plants was already observed in terms of enhanced antioxidant enzymes activities and higher production of MDA in *A. bettzickiana* by Tauqeer et al. [15], in *B. napus* by Shakoor et al. [24], and in *S. nigrum* by Ji et al. [31]. Kanwal et al. [14] reported similar results for *A. bettzickiana* when exposed to Cd stress. Addition of AA with Pb improved the plant defensive mechanisms which helped to overcome the lipid peroxidation caused by Pb stress. A similar mechanism was reported by Kanwal et al. [14] in *A. bettzickiana* under Cd and citric acid treatment. Some antioxidant enzymes (SOD, CAT, and APX), as well as other metabolites, perform a specific role in the tolerance and adaptation of plants to Pb ecotoxicity [4]. At 10 mM Pb, the antioxidant enzymatic activities tended to decrease while production of MDA continued to increase, which resulted in disruption of plant metabolic pathways and reduced nutrient uptake [14,29]. Enhanced production of MDA is usually observed as an indication of severe oxidative stress under metal stress which eventually destroys the plant cells [32,33].

### 3.4. Lead Accumulation, Concentration, and Translocation Factor

Accumulation of HMs in plant tissues is evidently associated with their concentration in the environment. In the present study, the Pb concentration in all parts of *A. bettzickiana* increased with increasing concentration of applied Pb in soil. The larger uptake of Pb in roots compared to leaves and stems was due to the direct exposure of roots to Pb in soil [34]. Our findings agree with those of Tauqeer et al. [15] and Kanwal et al. [14], who confirmed the phytoextraction potential of *A. bettzickiana* for Pb and Cd, respectively. However, a few studies suggested that Pb mostly accumulated in the roots, and only a small fraction can be translocated to the aerial parts of plants [35]. Similar to other plants such as *B. napus*, *L. minor,* and *Typha latifolia*, [24,26], *A. bettzickiana* accumulated Pb from media [15]. Conversely, a few plant species such as *T. orientali* restricted the accumulation of Pb in roots [34]. Addition of AA under Pb stress significantly increased the uptake of Pb and its accumulation in leaves, stems, and roots, similar to the findings reported by Bjelkova et al. [36] and Ji et al. [31]. AA and other organic acids offer the electrons and protons and construct complex ions to be readily up taken by plant roots [37]. Ji et al. [27] confirmed that indole-3-acetic acid significantly enhanced the uptake of Pb, Cd, and Zn in *S. nigrum*. In present study, the TF was observed <1 at all Pb concentrations with and without AA amendment. The similar results have been reported by Suthari et al. [38] for Alternanthera plant species such as A. philoxeroides for Pb at 9 different sites ranges from 0.35–0.96 which was higher than the TF of Fe and Mn and lower than Zn.

## 4. Materials and Methods

*A. bettzickiana* plants and the surrounding loamy soil were collected from the botanical garden of the University of Gujrat, Gujrat, Pakistan. The physico-chemical parameters of soil are listed in Table 4. A pot-based experiment was conducted in the Botanical Garden of University of Gujrat under natural environmental conditions to evaluate the effect of Pb on the growth of *A. bettzickiana* and its uptake under acetic acid amendments.

### 4.1. Growth Conditions

All plants were cleaned with distilled water and cuttings were planted individually in pots. All pots were watered regularly with tap water to sustain soil moisture. After 20 days of growth, each pot had a healthy plant and was allowed to grow for further 3 weeks before the application of Pb stress. The experiment was performed from March to May 2017.

### 4.2. Treatments

After 6 weeks of cultivation, different Pb concentrations were applied to plants alone and in combination with AA: T1, CK (control plant without Pb and AA); T2, AA 2.5 mM; T3, Pb 2.5 mM; T4, Pb 2.5 mM + AA 2.5 mM; T5, Pb 5 mM; T6, 5 mM + AA 2.5 mM; T7, Pb 7.5 mM; T8, 7.5 mM + AA 2.5 mM; T9, Pb 10 mM; and T10, Pb 10 mM + AA 2.5 mM. The Pb treatments were prepared from the most appropriately available lead nitrate (Pb(NO_3_)_2_. A dose of 250 mL for each treatment of Pb was applied weekly. However, in combined treatments (Pb + AA), a total of 500 mL was applied weekly for the next 4 weeks. A completely randomized design (CRD) for the experiment was followed with three replicates of each treatment.

### 4.3. Experiment Duration and Harvesting

After 4 weeks of treatment application, plants were harvested and carefully segregated into roots, leaves, and stems to measure agronomic traits such as height, number of leaves per plant (all leaves were included), and the fresh and dry biomass of plants. For dry biomass measurements, fresh samples of plant organs were placed in an oven for 72 h at 90 °C.

### 4.4. Leaf Area

Leaf area of plants was measured by using a leaf meter (L1-2000, LI-COR, Lincoln, NE, USA).

### 4.5. Determination of SPAD Value and Soluble Protein Content

The soil plant analysis development (SPAD) value/greenness of leaf was estimated using a SPAD-502 m (Zhejiang Top Instruments Co., Ltd., Zhejiang, Hangzhou, China). The fresh leaves and roots (0.5 g each) were ground using a prechilled mortar and pestle and then placed in 0.05 M phosphate buffer (pH 7.8). The mixture was then filtered through four layers of muslin cloth and centrifuged at 4 °C at 12,000× *g* for 10 min. The soluble protein content was estimated using Coomassie brilliant blue G-250 as a dye and albumin as a standard according to the Bradford method [39] using a UV-visible spectrophotometer (T60, PG Instruments, Warwick, UK).

### 4.6. Determination of Chlorophyll Contents

The fully-grown topmost leaves were separated to measure chlorophyll a, chlorophyll b, and carotenoids using a UV-visible spectrophotometer (T60, PG Instruments, Warwick, UK) according to the method reported by Metzner et al. [40]. Carotenoids contents and chlorophyll a and b contents were calculated using the following equations:

Chlorophyll a (µg mL^−1^) = 10.3 × E663 − 0.98 × E644, (1)

Chlorophyll b (µg mL^−1^) = 19.7 × E644 − 3.87 × E663, (2)

Total chlorophyll = chlorophyll a + chlorophyll b,(3)

Total carotenoids (µg mL^−1^) = 4.2 × E452.5 − ((0.0264 × chl a) + (0.426 × chl b)). (4)

At last, these pigment fractions were measured as mg g^−1^ fresh weight.

### 4.7. Determination of CAT, APX and MDA Contents

Antioxidant enzymes, such as catalase (CAT) and ascorbate peroxidase (APX), in roots and leaves were evaluated using a UV-visible spectrophotometer (T60, PG Instruments, Warwick, England). The activities of CAT and APX were estimated following the protocol reported by Aebi [41]. Similarly, malondialdehyde (MDA) concentration was measured by the thiobarbituric acid (TBA) reaction method of Heath and Packer [42] with minor modifications as proposed by Dhindsa et al. [43] and Zhang and Kirham [44].

### 4.8. Determination of Pb Content

We burned 0.5 g dry weight of the plant organ (root, stem, or leaf) to ash by placing it in a muffle furnace (Forno Vulcan 3-550, Dentsplay, York, PA, USA) at 650 °C for 7 h. The ash was mixed in concentrated hydrochloric acid (HCl) and nitric acid (HNO_3_) in a 1:3 ratio and placed on an orbital shaker for 30 min until the ash dissolved in solution. Finally, the volume was brough up to 50 mL by adding distilled water and examined using an atomic absorption spectrometer (NOVA A400, Analytik Jena, Jena, Germany) to measure Pb concentration as described by Ehsan et al. [45]. The Pb concentration was calculated as follows:

Pb concentration (mg kg^−1^) = Pb reading of digested sample (mg L^−1^) × dilution factor. (5)

The Pb accumulation was calculated as

Pb accumulation (mg plant^−1^) = Pb concentration in tissue (mg kg^−1^) × dry weight of plant organ (kg). (6)

The root to shoot translocation factor (TF) of *A. bettzickiana* was calculated as

TF = Metal concentration in aerial parts/Metal concentration in roots.(7)

### 4.9. Statistical Analysis

The data presented in this study are the average of three replicates for each treatment. ANOVA was performed followed by Tukey’s post-hoc test, and significant differences were calculated by all pairwise comparison by using statistical package SPSS version 16.0 (SPSS, Chicago, IL, USA). Furthermore, *t*-test was performed to determine the significant differences between treatments with and without AA for each soil Pb concentration. The different small letters in figures and tables describe values that are significantly different at *p* ≤ 0.05 and *** indicate significance at *p* < 0.01.

## 5. Conclusions

Pb ecotoxicity significantly decreased plant growth, photosynthetic pigments, plant biomass, protein content and antioxidants enzymes by increasing the lipid peroxidation along with higher Pb uptake. Acetic acid alleviated Pb ecotoxicity and enhanced growth attributes of the plant. Therefore, AA addition promoted photosynthetic attributes by stabilizing the oxidative damage of plant cells. AA promoted antioxidative defense systems of plants and reduced the production of ROS to decrease Pb ecotoxicity. The results also revealed that *A. bettzickiana* absorbed and accumulated larger amount of Pb in its roots, stems and leaves under AA and Pb treated plants as compared to Pb only treated plants. The present study also encouraged future endeavors to investigate the effects of Pb at plant genetic level along with the identification of plant detoxification mechanism.

## Figures and Tables

**Table 1:** Effect of Pb (0, 2.5, 5, 7.5 and 10 mM) with and without AA (2.5 mM) on fresh and dry biomass of leaf, stem and root of *A. bettzickiana*.

	Pb Concentration (mM)				
Treatments	Pb 0	Pb 2.5	Pb 5	Pb 7.5	Pb 10
	Root Dry Weight (g)				
AA 0	13.45 ± 0.89 ^c^	12.27 ± 0.32 ^c^	9.98 ± 0.41 ^ef^	8.25 ± 0.36 ^fg^	7.29 ± 0.44 ^g^
AA 2.5 mM	18.89 ± 0.40 ^a^	16.29 ± 0.81 ^b^	11.74 ± 0.38 ^cd^	10.26 ± 0.80 ^de^	9.65 ± 0.79 ^ef^
	Stem Dry Weight (g)				
AA 0	21.85 ± 0.70 ^b^	17.98 ± 0.94 ^c^	13.16 ± 0.57 ^d^	8.84 ± 0.39 ^e^	6.50 ± 0.38 ^f^
AA 2.5 mM	24.96 ± 0.84 ^a^	23.31 ± 0.64 ^ab^	19.24 ± 0.66 ^c^	13.03 ± 0.91 ^d^	10.75 ± 0.49 ^e^
	Leaf Dry Weight (g)				
AA 0	20.27 ± 0.95 ^bc^	18.18 ± 0.84 ^d^	14.07 ± 0.36 ^e^	11.26 ± 0.48 ^f^	9.50 ± 0.44 ^f^
AA 2.5 mM	23.22 ± 0.87 ^a^	20.88 ± 0.65 ^b^	18.87 ± 0.77 ^cd^	17.41 ± 0.43 ^d^	13.39 ± 0.55 ^e^
	Root Fresh Weight (g)				
AA 0	12.55 ± 0.56 ^de^	10.63 ± 0.25 ^f^	8.48 ± 0.21 ^g^	9.21 ± 0.22 ^g^	7.28 ± 0.28 ^h^
AA 2.5 mM	17.37 ± 0.30 ^a^	15.27 ± 0.27 ^b^	14.00 ± 0.58 ^c^	13.18 ± 0.65 ^cd^	11.41 ± 0.28 ^ef^
	Stem Fresh Weight (g)				
AA 0	22.99 ± 0.82 ^a^	18.90 ± 0.78 ^b^	14.19 ± 0.60 ^cd^	9.85 ± 0.38 ^e^	7.50 ± 0.38 ^f^
AA 2.5 mM	24.29 ± 0.58 ^a^	23.02 ± 0.89 ^a^	20.24 ± 0.66 ^b^	15.96 ± 0.81 ^c^	12.28 ± 0.72 ^d^
	Leaf Fresh Weight (g)				
AA 0	21.60 ± 0.42 ^bc^	20.50 ± 0.50 ^cd^	17.39 ± 0.29 ^e^	14.59 ± 0.30 ^f^	11.61 ± 0.39 ^g^
AA 2.5 mM	25.45 ± 0.42 ^a^	22.56 ± 0.42 ^b^	20.47 ± 0.50 ^cd^	19.53 ± 0.41 ^d^	16.43 ± 0.35 ^e^
Treatments	***	***	***	***	***
Pb	***	***	***	***	***
Tr × Pb	***	***	***	***	***

Values are mean of three replicates ± S.D. Different lowercase letters indicate significant differences between treatments at *p* < 0.05; and *** indicate significance at the *p* < 0.01 level.

**Table 2:** Effect of Pb (0, 2.5, 5, 7.5 and 10 mM) with and without AA (2.5 mM) on growth parameters of *A. bettzickiana*.

	Pb Concentration (mM)				
Treatments	Pb 0	Pb 2.5	Pb 5	Pb 7.5	Pb 10
	Plant Height (cm)				
AA 0	15.57 ± 0.34 ^c^	13.57 ± 0.46 ^d^	12.52 ± 0.20 ^e^	11.78 ± 0.26 ^e^	10.58 ± 0.19 ^f^
AA 2.5 mM	19.52 ± 0.36 ^a^	17.53 ± 0.40 ^b^	16.60 ± 0.51 ^b^	14.08 ± 0.32 ^d^	12.55 ± 0.18 ^e^
	Root Length (cm)				
AA 0	24.97 ± 0.83 ^bc^	23.17 ± 1.05 ^cde^	22.43 ± 1.09 ^de^	21.41 ± 0.34 ^ef^	19.56 ± 0.36 ^f^
AA 2.5 mM	32.44 ± 1.17 ^a^	26.55 ± 0.38 ^b^	24.30 ± 0.39 ^bcd^	23.84 ± 0.55 ^cd^	21.27 ± 1.19 ^ef^
	Leaf Area (cm^2^)				
AA 0	8.71 ± 0.19 ^b^	8.25 ± 0.38 ^bc^	6.91 ± 0.31 ^de^	6.47 ± 0.35 ^def^	5.33 ± 0.20 ^g^
AA 2.5 mM	12.82 ± 0.46 ^a^	9.22 ± 0.50 ^b^	7.41 ± 0.38 ^cd^	6.19 ± 0.31 ^efg^	5.74 ± 0.15 ^fg^
	No. of Leaves Plant^−1^				
AA 0	1259.33 ± 40.01 ^c^	1055.66 ± 20.09 ^de^	861.66 ± 33.65 ^fg^	834.33 ± 35.11 ^g^	672.00 ± 45.13 ^h^
AA 2.5 mM	1649.66 ± 37.55 ^a^	1452.33 ± 49.08 ^b^	1159.33 ± 29.56 ^cd^	954.33 ± 32.08 ^ef^	810.00 ± 40.03 ^g^
Treatments	***	***	***	***	***
Pb	***	***	***	***	***
Tr × Pb	***	***	***	***	***

Values are mean of three replicates ± S.D. Different lowercase letters indicate significant differences between treatments at *p* < 0.05; and *** indicate significance at the *p* < 0.01 level.

**Table 3:** Effect of Pb (0, 2.5, 5, 7.5 and 10 mM) with and without AA (2.5 mM) on Pb concentration, accumulation and root-shoot translocation factor (TF) in *A. bettzickiana*.

Pb Concentration (mg kg^−1^)				Pb Accumulation (µg Plant^−1^)			TF
Treatments	Leaf	Stem	Root	Leaf	Stem	Root	
**CK**	0.01 ± 0.00 ^h^	0.06 ± 0.08 ^h^	0.06 ± 0.02 ^i^	0.15 ± 0.001 ^f^	1.28 ± 0.06 ^f^	0.84 ± 0.01 ^h^	0.87 ± 0.75 ^a^
**AA**	0.02 ± 0.00 ^g^	0.02 ± 0.00 ^h^	1.74 ± 0.18 ^i^	0.37 ± 0.001 ^f^	0.58 ± 0.003 ^f^	32.86 ± 0.07 ^g^	0.02 ± 0.00 ^b^
**Pb 2.5 mM**	4.16 ± 0.96 ^f^	7.04 ± 2.04 ^g^	13.95 ± 3.91 ^h^	75.56 ± 0.73 ^e^	126.60 ± 1.73 ^e^	171.30 ± 1.64 ^f^	0.80 ± 0.04 ^a^
**Pb 2.5 + AA**	12.33 ± 0.90 ^e^	15.54 ± 1.24 ^f^	30.48 ± 2.05 ^g^	257.50 ± 0.31 ^c^	362.47 ± 0.71 ^c^	496.82 ± 1.69 ^e^	0.91 ± 0.02 ^a^
**Pb 5 mM**	13.18 ± 0.98 ^de^	18.48 ± 0.85 ^f^	36.24 ± 1.84 ^f^	185.48 ± 0.31 ^d^	243.19 ± 0.48 ^d^	361.63 ± 0.80 ^e^	0.87 ± 0.03 ^a^
**Pb 5 + AA**	15.87 ± 1.51 ^d^	22.54 ± 2.42 ^e^	45.06 ± 5.11 ^e^	299.52 ± 1.05 ^c^	433.69 ± 1.60 ^b^	528.95 ± 1.48 ^d^	0.85 ± 0.05 ^a^
**Pb 7.5**	17.45 ± 1.06 ^d^	30.50 ± 1.41 ^d^	62.56 ± 2.29 ^d^	196.48 ± 0.44 ^d^	269.91 ± 0.47 ^d^	516.11 ± 0.82 ^d^	0.76 ± 0.06 ^a^
**Pb 7.5 + AA**	21.67 ± 1.20 ^c^	34.90 ± 1.21 ^c^	72.74 ± 2.66 ^c^	377.27 ± 0.59 ^b^	454.72 ± 1.01 ^b^	746.27 ± 1.88 ^b^	0.77 ± 0.01 ^a^
**Pb 10**	26.96 ± 2.62 ^b^	38.39 ± 1.10 ^b^	83.68 ± 2.84 ^b^	256.42 ± 1.33 ^c^	249.90 ± 0.40 ^d^	610.03 ± 1.13 ^c^	0.78 ± 0.00 ^a^
**Pb 10 + AA**	32.48 ± 2.17 ^a^	48.14 ± 2.17 ^a^	95.34 ± 1.27^a^	434.95 ± 1.12 ^a^	517.48 ± 0.89 ^a^	919.99 ± 0.91 ^a^	0.84 ± 0.04 ^a^
*Treatments*	***	***	***	***	***	***	***
*Pb*	***	***	***	***	***	***	***
*Tr × Pb*	***	***	***	***	***	***	***

Values are mean of three replicates ± S.D. Different lowercase letters indicate significant differences between treatments at *p* < 0.05; and *** indicate significance at the *p* < 0.01 level.

**Table 4:** Physico-chemical characterization of soil.

Soil Properties	
Texture	60 (loam)
Saturation (%)	35
pH	7.9
EC (μS cm^−1^)	1401
Organic matter (%)	0.46–0.59
Available Phosphorus (mg kg^−1^)	5
Total Nitrogen (%)	0.037
Exchangeable Sodium (mMc 100 g^−1^)	0.7
Potassium (mg kg^−1^)	200
Calcium Carbonate (mg kg^−1^)	0.1
HCO3 (mmol L^−1^)	2.40
Cl- (mmol L^−1^)	1.69
Available Pb (mg kg^−1^)	0.01
